# Inhibition of invasion and induction of apoptotic cell death of cancer cell lines by overexpression of TIMP-3

**DOI:** 10.1038/sj.bjc.6690217

**Published:** 1999-03

**Authors:** A H Baker, S J George, A B Zaltsman, G Murphy, A C Newby

**Affiliations:** Division of Surgery, University of Bristol, Bristol Royal Infirmary, Bristol, BS2 8HW, UK; School of Biological Sciences, University of East Anglia, Norwich, NR4 7TJ, UK

**Keywords:** tissue inhibitor of metalloproteinase, cancer cell, apoptosis, cell invasion, adenovirus, gene therapy

## Abstract

Dysregulation of matrix degrading metalloproteinase enzymes (MMPs) leads to increased extracellular matrix turnover, a key event in the local invasion and metastasis of many tumours. The tissue inhibitors of metalloproteinases (TIMPs) limit the activity of MMPs, which suggests their use in gene therapy. We have previously shown that overexpression of TIMP-1, -2 or -3 inhibits vascular smooth muscle and melanoma cell invasion, while TIMP-3 uniquely promotes apoptosis. We have therefore sought to determine whether TIMP-3 can inhibit invasion and promote apoptosis in other cancer cell types. Adenoviral-mediated overexpression of TIMP-3 inhibited invasion of HeLa and HT1080 cells through artificial basement membrane to similar levels as that achieved by TIMP-1 and -2. However, TIMP-3 uniquely promoted cell cycle entry and subsequent death by apoptosis. Apoptosis was confirmed by morphological analysis, terminal dUTP nick end labelling (TUNEL) and flow cytometry. The apoptotic phenotype was mimicked by addition of exogenous recombinant TIMP-3 to uninfected cultures demonstrating that the death signal is initiated extracellularly and that a bystander effect exists. These results show that TIMP-3 inhibits invasion in vitro and promotes apoptosis in cancer cell type of differing origin. This clearly identifies the potential of TIMP-3 for gene therapy of multiple cancer types. © 1999 Cancer Research Campaign


*British Journal of Cancer* (1999) **79**(9/10), 1347–1355
© 1999 Cancer Research Campaign
Article no. bjoc.1998.0217

### Inhibition of invasion and induction of apoptotic cell death of cancer cell lines by overexpression of TIMP-3

**AH Baker[1], SJ George[1], AB Zaltsman[1], G Murphy[2] and AC Newby[1]**

[1]University of Bristol, Division of Surgery, Level 7, Bristol Royal Infirmary, Bristol BS2 8HW, UK; [2]School of Biological Sciences, University of East Anglia, Norwich NR4 7TJ, UK

**Summary** Dysregulation of matrix degrading metalloproteinase enzymes (MMPs) leads to increased extracellular matrix turnover, a key event in the local invasion and metastasis of many tumours. The tissue inhibitors of metalloproteinases (TIMPs) limit the activity of MMPs, which suggests their use in gene therapy. We have previously shown that overexpression of TIMP-1, -2 or -3 inhibits vascular smooth muscle and melanoma cell invasion, while TIMP-3 uniquely promotes apoptosis. We have therefore sought to determine whether TIMP-3 can inhibit invasion and promote apoptosis in other cancer cell types. Adenoviral-mediated overexpression of TIMP-3 inhibited invasion of HeLa and HT1080 cells through artificial basement membrane to similar levels as that achieved by TIMP-1 and -2. However, TIMP-3 uniquely promoted cell cycle entry and subsequent death by apoptosis. Apoptosis was confirmed by morphological analysis, terminal dUTP nick end labelling (TUNEL) and flow cytometry. The apoptotic phenotype was mimicked by addition of exogenous recombinant TIMP-3 to uninfected cultures demonstrating that the death signal is initiated extracellularly and that a bystander effect exists. These results show that TIMP-3 inhibits invasion in vitro and promotes apoptosis in cancer cell type of differing origin. This clearly identifies the potential of TIMP-3 for gene therapy of multiple cancer types.

**Keywords:** tissue inhibitor of metalloproteinase; cancer cell; apoptosis; cell invasion; adenovirus; gene therapy

The extracellular matrix is an important structure in maintenance of tissue organization and in the suppression of cellular proliferation and migration. Excess destruction of the extracellular matrix is associated with many pathologies including atherosclerosis, rheumatoid arthritis and cancer progression. The intricate balance between net extracellular matrix deposition and degradation is controlled by a complex system of tightly regulated protease enzymes and their endogenous inhibitors. Matrix destruction is thought to be a key event in both the local invasion and distant metastasis associated with tumour progression and genes that inhibit these processes may be useful for cancer therapy.

The matrix degrading metalloproteinases (MMPs) are a large family of enzymes that together can degrade all components of the extracellular matrix (Birkedal-Hansen et al, 1993). Within the extracellular matrix, the tissue inhibitor of metalloproteinases (TIMPs), of which there are four family members (Docherty et al, 1985; Stetler-Stevenson et al, 1990; Uría et al, 1994; Leco et al, 1997), inhibit the activity of MMPs by binding with a 1:1 stoichiometry to the active site (Bode et al, 1994). Proteolytic processing of pro-MMP-9 to its active form is also inhibited by additional binding of TIMP-1 at the C-terminal site, while TIMP-2 plays a more complex biphasic role in the activation of MMP-2 (Murphy et al, 1992; Strongin et al, 1995). A number of studies have indicated the potential use of overexpression of TIMP genes for cancer therapy using plasmid, and retrovirus-based gene transfer systems (DeClerck et al, 1992; Imren et al, 1992; Montgomery et al, 1994; Watanabe et al, 1996; Smith et al, 1997; Wang et al, 1997), or by using synthetic MMP inhibitors (Koop et al, 1994; Wang et al, 1994; Sledge et al, 1995; Low et al, 1996; Watson et al, 1996).

Unlike TIMP-1, -2 and -4, TIMP-3 is sequestered into the extracellular matrix (Leco et al, 1994) although the precise binding site(s) for TIMP-3 are not yet known. High levels of TIMP-3 are expressed in cartilage, epithelia, muscle and in invading cytotrophoblasts (Apte et al, 1994; Bass et al, 1997) and have been detected in breast carcinoma (Uría et al, 1994) localized to the stroma (Byrne et al, 1995). Our previous studies have shown that overexpression of TIMP-3 in vascular smooth muscle cells and melanoma cell lines inhibits invasion and promotes apoptotic cell death (Ahonen et al, 1998; Baker et al, 1998). To investigate the potential use of TIMP-3 for gene therapy of cancer of different cell types, we have used a replication-defective recombinant-adenovirus capable of overexpressing TIMP-3 (Baker et al, 1998). We have assessed phenotypic changes associated with TIMP-3 overexpression on non-invasive MCF-7 adenocarcinoma cells (Noel et al, 1991), moderately invasive HeLa cervical carcinoma cells (Nuovo, 1997) and highly invasive HT1080 fibrosarcoma cells (Noel et al, 1991) following adenoviral infection in vitro.

### MATERIALS AND METHODS

### Materials

All chemical reagents were obtained from Sigma (Poole, Dorset, UK) unless otherwise stated. Cell culture reagents were obtained from Gibco/BRL (Paisley, UK). P231 (aka BB-94) was a generous gift from Pfizer (Sandwich, Kent, UK). The replication-defective recombinant adenoviruses RAd35 (expresses lacZ from the CMV

*Received 25 June 1998*
*Revised 30 September 1998*
*Accepted 1 October 1998*

*Correspondence to:* AH Baker

IEP), RAd66 (expresses no transgene but contains the CMV IEP polyadenylation signal), RAdTIMP-1, -2 and -3 (express TIMP-1, -2, or -3 from the CMV IEP, respectively) have been described in detail elsewhere (Wilkinson and Akrigg, 1992; Baker et al, 1996, 1998). Stocks of adenovirus were purified on caesium chloride gradients and assessed for lack of replication competent adenovirus by titration on non-permissive HeLa cells.

### METHODS

### Cell culture

HeLa, MCF-7 and HT1080 cells were cultured in Dulbecco's modified Eagle's medium (DMEM) containing 10% fetal calf serum (FCS), 100 μg/ ml$^{-1}$ penicillin and 100 units per ml streptomycin (complete media) unless otherwise stated and maintained in a humidified atmosphere at 37°C in 5% carbon dioxide. For assays, $1 \times 10^5$ cells were plated in 6-well plates 24 h prior to infection (for certain experiments, cells were plated in a similar manner but onto glass coverslips). One set of triplicate wells were counted for an accurate calculation of cell number immediately prior to infection. Cells were incubated with recombinant adenoviruses in 2 ml of fresh complete media and left for 18 h. Cells were then washed and further incubated in fresh complete media until the required time point.

### Quantification of adenoviral infection efficiency

Cells were either mock infected or infected with RAd35 at increasing multiplicities of infection (MOI) for 18 h, washed and incubated for a further 24 h in complete media. Cells were stained with X-gal stain (5-bromo-4-chloro-3-indoyl-β-D-galactopyranosidase) [100 mM sodium phosphate pH 7.3 (77 mM $Na_2HPO_4$, 23 mM $NaH_2PO_4$), 1.3 mM $MgCl_2$, 3 mM $K_3Fe(CN)_6$, 3 mM $K_4Fe(CN)_6$ and 1 mg ml$^{-1}$ X-gal] and counterstained with nuclear fast red. Positive (blue cells) were counted in four high power fields per section from triplicate cultures and the percentage infection efficiency calculated.

### Analysis of transgene production

HeLa cells were used for analysis of TIMP-3 overexpression. Briefly, cells were infected for 18 h in complete media, washed and further incubated in media containing 2% FCS for 24 h. For all infections, the conditioned media (2 ml) were collected and stored at –20°C prior to analysis. As TIMP-3 is associated with the extracellular matrix (Leco et al, 1994), the cell and extracellular matrix fraction was harvested from uninfected, RAd66 and RAdTIMP-3 infected cells as described elsewhere (Baker et al, 1998). Conditioned media were concentrated tenfold using Centricon microconcentrators (Amicon Inc., Stonehouse, UK). All samples were electrophoresed on 11% polyacrylamide gels and membranes were probed with a mouse anti-human TIMP-3 (a generous gift from K Iwata, Fuji Chemicals Ltd, Japan). Bands were visualized using enhanced chemiluminescence (ECL, Amersham International, UK).

### Matrigel invasion assay

Infected and uninfected HeLa and HT1080 cells were analysed for their invasive capacity across artificial basement membrane using modified Boyden chambers (Nucleopore, Middlesex, UK). Briefly, $2 \times 10^4$ HeLa cells (or $5 \times 10^3$ HT1080 cells) in DMEM without phenol red containing 1% bovine serum albumin (BSA) were placed in the upper chamber with a 0.4 μg/mm$^2$ Matrigel barrier (Stratech, Luton, UK). Media containing 10% FCS as a chemoattractant were placed in the lower chamber and invasion allowed to commence for 24 h. Uninfected cells were also incubated with BB-94 to compare TIMP-3 effects with those of a synthetic MMP inhibitor. Cells that had invaded the basement membrane were fixed in methanol, stained with haematoxylin and cells on the lower surface of the filter counted ($4 \times 200$ fields per section).

### Analysis of cell number

At the required time point, viable adherent cells from triplicate cultures were counted using a haemacytometer and trypan blue exclusion.

### Terminal dUTP nick end labelling (TUNEL) analysis of cell death by apoptosis

Following fixation in methanol, HeLa cells were washed in $1 \times$ TE (10 mM Tris-HCl, 1 mM EDTA, pH 8.0) and labelled in buffer containing 100 mM cacodylate (pH 6.4), 1 mM $CoCl_2$, 1 mM dithothreitol, 0.01 mM biotin-dUTP and 8 units per ml terminal deoxynucleotidyl transferase (Promega, Southampton, UK) at 37°C for 60 min. Cells were washed, and biotin-dUTP labelled nuclei were detected using streptavidin–peroxidase and diaminobenzidine staining.

### Evaluation of bromodeoxyuridine (BrdU) incorporation

Cells were pulsed with 10 μM BrdU for 1 h prior to fixation. Cells were stained using a mouse monoclonal anti-BrdU antibody and immunocytochemical detection. Cells were counted in four high-powered fields per section from triplicate cultures.

### Flow cytometric analysis of apoptosis and cell cycle parameters

Cells were trypsinized at the required time point, washed, resuspended in phosphate-buffered saline (PBS) and fixed in 70% ethanol in PBS. Prior to analysis, cells were resuspended in 400 μl of PBS, and 50 μl of 400 μg ml$^{-1}$ propidium iodine and 50 μl of 100 μg ml$^{-1}$ RNaseA were added and left at room temperature for 30 min. Cells were analysed on a Becton-Dickinson FACScan flow cytometer and data analysed using winMDI and Multicycle software.

### Statistical analysis

All data were analysed using an unpaired Student's *t*-test and are shown as the mean value ± s.e.m.

### RESULTS

### Adenoviral infection efficiency

In uninfected HeLa, HT1080 and MCF-7 cells, no β-galactosidase positivity was observed (not shown). HeLa cells were efficiently

 © Cancer Research Campaign 1999

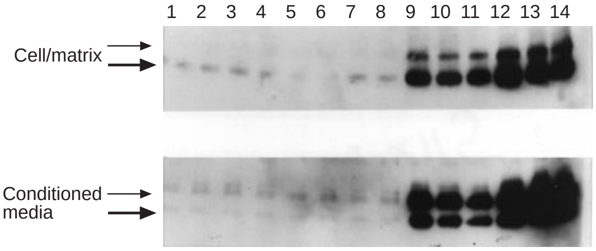

**Figure 1** Analysis of recombinant TIMP-3 secretion. A total of $10^5$ HeLa cells were infected with recombinant adenoviruses for 18 h, washed and maintained in 2% FCS-containing media for 24 h. Conditioned media (tenfold concentrated) and cell/matrix lysates were subsequently analysed for TIMP-3 by Western blot analysis. 1, 2: uninfected control cells; 3–5: RAd66 30 pfu per cell; 6–8: RAd66 80 pfu per cell; 9–11: RAdTIMP-3 30 pfu per cell; 12–14: RAdTIMP-3 80 pfu per cell. The small arrow represents the glycosylated form of TIMP-3 and the large arrow the unglycosylated form

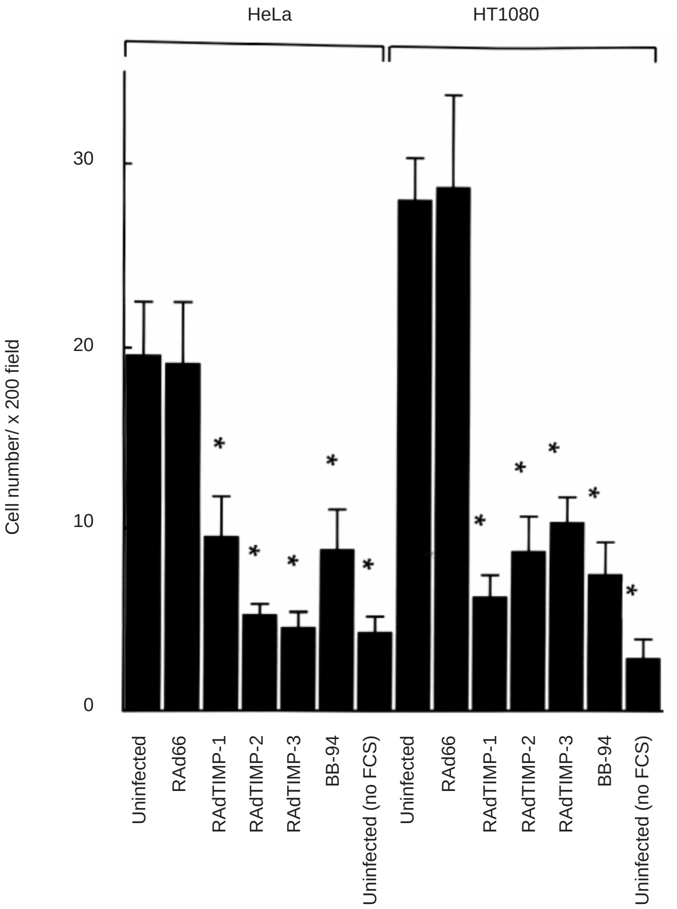

**Figure 2** Effect of TIMP-3 on cancer cell invasion in vitro. Cells were infected with recombinant adenoviruses at 80 pfu per cell for HeLa and 300 pfu per cell for HT1080 cells for 18 h. A total of $2 \times 10^4$ HeLa cells or $5 \times 10^3$ HT1080 cells were then analysed for invasion through reconstituted basement membrane following overexpression of individual TIMPs. *Indicates statistical significance ($P < 0.05$, $n = 12$) vs uninfected and RAd66-infected controls

© Cancer Research Campaign 1999 

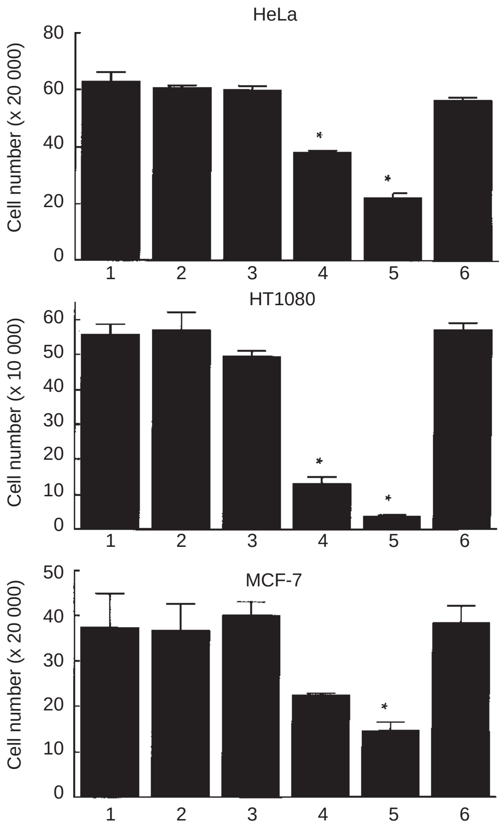

**Figure 3** Effect of TIMP-3 overexpression on cancer cell growth. A total of $1 \times 10^5$ HeLa, HT1080 or MCF-7 cells were infected with RAd66 or RAdTIMP-3 and cultured in complete media until 90 h post infection (66 h for HT1080 cells). Viable adherent cells were counted from triplicate cultures. 1: uninfected cells; 2 and 3: RAd66-infected cells (30 and 80 pfu per cell for HeLa cells; 100 and 300 pfu per cell for HT1080 cells; 200 and 600 pfu per cell for MCF-7 cells, respectively); 4 and 5: RAdTIMP-3-infected cultures at same pfu per cell as (2) and (3), respectively; 6: BB-94. *Indicates statistical significance ($P < 0.05$ vs respective RAd66 control, $n = 3$)

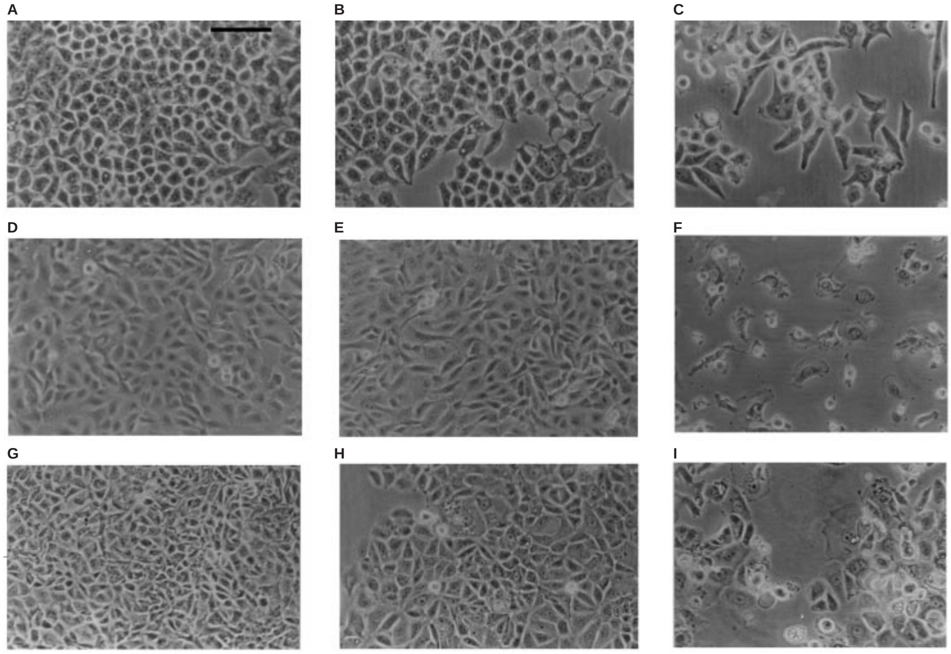

**Figure 4** Morphological analysis of cultures. Phase contrast photographs of HeLa (**A**–**C**), HT1080 (**D**–**F**) and MCF-7 (**G**–**I**) cells at 66 h post infection. Uninfected (**A, D, G**), RAd66 control adenovirus-infected (**B**, 80 pfu per cell; **E**, 300 pfu per cell; **H**, 600 pfu per cell) and RAdTIMP-3-infected (**C**, 80 pfu per cell; **F**, 300 pfu per cell; **I**, 600 pfu per cell) cells are shown. The scale bar in **A** represent 50 μm and is applicable to all panels

 *© Cancer Research Campaign 1999*

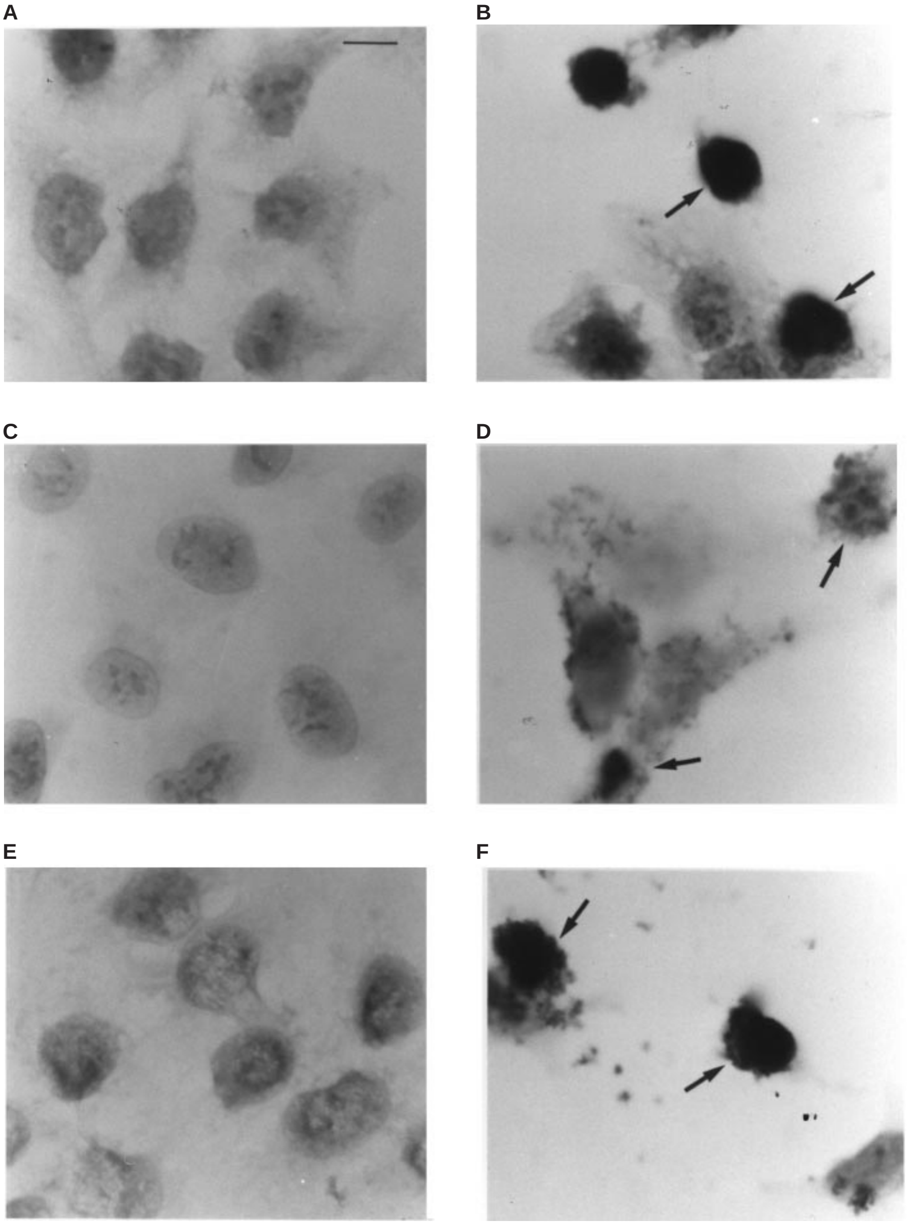

**Figure 5** TUNEL analysis of cancer cells. HeLa (**A** and **B**, 80 pfu per cell), HT1080 (**C** and **D**, 300 pfu per cell) and MCF-7 cells (**E** and **F**, 600 pfu per cell) were analysed 66 h post infection for TUNEL positivity as a marker of apoptosis following infection with RAd66 (**A, C, E**) or RAdTIMP-3 (**B, D, F**). Dark nuclei represent TUNEL-positive cells (representative examples indicated by arrows in **B**, **D** and **F**). The scale bar in **A** represents 10 μm and is applicable to all panels and dose-dependently infected with RAd35 as assessed by staining for β-galactosidase. Infection with 30 and 80 pfu per cell resulted in 35 ± 1% and 70 ± 1% infection, respectively. Higher MOIs were required for high level infection of HT1080 and MCF-7 cells (HT1080 cells: 100 pfu per cell 62 ± 3%, 300 pfu per cell 94 ± 2%; MCF-7 cells: 200 pfu per cell 35 ± 2%, 600 pfu per cell 66 ± 4%).

### Analysis of transgene production

Uninfected HeLa cells showed very low level production of TIMP-3 both in the cell/matrix fraction and in the conditioned media (Figure 1). RAd66 infection at 30 or 80 pfu per cell had no effect on TIMP-3 secretion per se (Figure 1). For RAdTIMP-3-infected cells, high level transgene production was observed both in the cell/matrix fraction and in the conditioned media consistent with previous observations (Figure 1; Baker et al, 1998). Both the unglycosylated and glycosylated form of TIMP-3 were detected (Figure 1). Western blot analysis of uninfected and RAd66-infected HT1080 and MCF-7 cell/matrix extracts failed to show the presence of endogenous TIMP-3 but RAdTIMP-3 infection evoked similar high level TIMP-3 production (not shown).

### TIMP-3 overexpression inhibits cancer cell invasion

To ascertain the effect of high level overexpression of TIMP-3 on invasion of cancer cells in vitro, modified Boyden chamber assays

© Cancer Research Campaign 1999

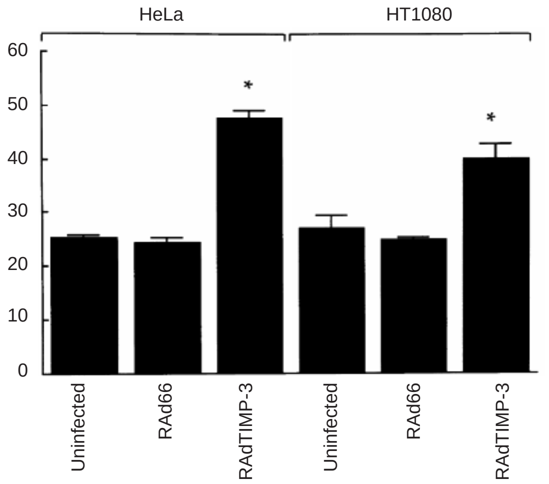

**Figure 6** Effect of TIMP-3 overexpression on BrdU incorporation. Uninfected and infected HeLa (80 pfu per cell) and HT1080 (300 pfu per cell) cells were pulsed with 10 μM BrdU for 1 h at 42 h post infection and the percentage BrdU positivity determined immunocytochemically from triplicate cultures. *Indicates statistical significance from the uninfected and RAd66 control adenovirus-infected culture ($P < 0.05$, $n = 3$).

**Table 1** Quantification of cell cycle characteristics of uninfected and infected HeLa and HT1080 cells

| Cell line | Treatment | $G_0/G$ | S | $G_2/M$ | [a]Pre-$G_0$ |
|---|---|---|---|---|---|
| HeLa | Uninfected | 55 ± 0.5 | 27 ± 0.3 | 18 ± 0.2 | 3 ± 0.2 |
| | RAd66 80 pfu per cell | 51 ± 0.3 | 28 ± 0.3 | 20 ± 1 | 3 ± 1 |
| | RAdTIMP-3 80 pfu per cell | 39 ± 1[b] | 35 ± 0.3[b] | 26 ± 1[b] | 9 ± 1[b] |
| HT1080 | Uninfected | 51 ± 2 | 28 ± 1 | 23 ± 1 | 5 ± 2 |
| | RAd66 100 pfu per cell | 53 ± 2 | 23 ± 2 | 25 ± 0.4 | 5 ± 1 |
| | RAd66 300 pfu per cell | 51 ± 1 | 22 ± 1 | 26 ± 0.2 | 5 ± 1 |
| | RAdTIMP-3 100 pfu per cell | 29 ± 3[b] | 34 ± 2[b] | 37 ± 1[b] | 9 ± 1[b] |
| | RAdTIMP-3 300 pfu per cell | 15 ± 0.2[b] | 47 ± 1[b] | 38 ± 1[b] | 12 ± 0.2[b] |

Cells were fixed 66 h post infection and analysed by propidium iodide staining and flow cytometric analysis for cell cycle populations following RAd66 and RAdTIMP-3 infection. [a]For pre-$G_0$ analysis, data are presented as the percentage of the total gated cell population. For $G_0/G_1$, S and $G_2/M$, data are calculated from the percentage of cells in cycle (i.e. excluding cells in the pre-$G_0$ region). [b]$P < 0.05$ for RAdTIMP-3- versus respective RAd66-infected control.

were performed on uninfected, RAd66- and RAdTIMP-3-infected cells. Due to the non-invasive capacity of MCF-7 cells (Nuovo, 1997), the effect of TIMP-3 overexpression on MCF-7 cell invasion was not analysed. For HeLa and HT1080 cells, infection with RAd66 (at 80 and 300 pfu per cell, respectively) had no effect on the invasion of either cell type compared to uninfected cells (Figure 2). Overexpression of TIMP-3 significantly inhibited invasion of HeLa and HT1080 cells through reconstituted basement membrane compared to uninfected and RAd66-infected controls (Figure 2). The effect of TIMP-3 was comparable to that observed by TIMP-1, TIMP-2 and the synthetic MMP inhibitor BB-94 (Figure 2).

### TIMP-3 overexpression induces a dose-dependent reduction in cell number

Infection of HeLa, HT1080 and MCF-7 cells with RAd66 had no significant effect on cell numbers at 90 h post infection (Figure 3). In contrast, TIMP-3 overexpression resulted in a dose-dependent reduction in cell number for each cell type (Figure 3). The effect of TIMP-3 was not mimicked when uninfected cells were cultured with BB-94 (Figure 3).

### TIMP-3 stimulates cell cycle entry and apoptosis in cancer cells

We previously demonstrated in vascular smooth muscle cells that overexpression of TIMP-3 enhanced DNA synthesis and induced apoptosis. Morphological analysis of HeLa, HT1080 and MCF-7 cultures postinfection revealed that TIMP-3 overexpression resulted in changes associated with apoptotic cell death including membrane blebbing, cell shrinkage and nuclear chromatin condensation (Figure 4). No evidence of cell death was observed with overexpression of TIMP-1 or -2 or by culture in the presence of BB-94 (results not shown). To evaluate apoptosis in TIMP-3 overexpressing cells further, TUNEL analysis was performed. While infection of cells with RAd66 or treatment with BB-94 had no significant effect on the number of TUNEL-positive cells (Figure 5, BB-94 not shown), RAdTIMP-3 infection resulted in high levels of TUNEL positive cells in HeLa, HT1080 and MCF-7 cells (Figure 5). In HeLa cells, TUNEL positivity increased from 2.7 ± 0.7% for uninfected controls, 3.1 ± 1.3% for RAd66-infected cells and 2.58 ± 0.7% for BB-94-treated cultures to 33.5 ± 0.6% for RAdTIMP-3-infected cells ($P < 0.05$ for RAdTIMP-3-infected HeLa cells vs uninfected, RAd66- and BB-94-treated cultures,

 © Cancer Research Campaign 1999

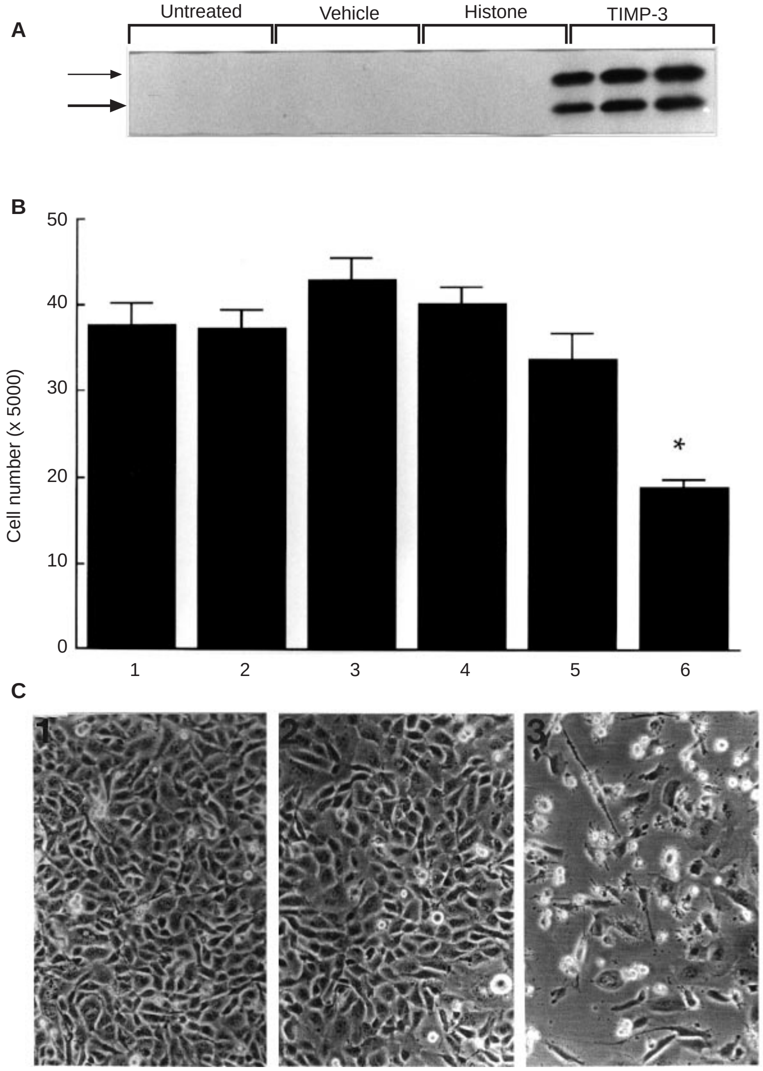

**Figure 7** Effect of exogenous recombinant TIMP-3 on uninfected cancer cells. A total of $2 \times 10^4$ uninfected HT1080 cells were cultured in the presence of vehicle, control protein (histone) or rTIMP-3. (**A**) Western blot analysis of cell/matrix lysates from cells 48 h post addition of histone or rTIMP-3 (both 2 μg). The large arrow represents the unglycosylated form of TIMP-3 and the small arrow, the glycosylated form. (**B**) At 48 h post addition, viable adherent cells were counted. 1: untreated control cells; 2: vehicle; 3 and 4: 0.5 and 2 μg histone, respectively; 5 and 6: 0.5 and 2 μg rTIMP-3, respectively. *Indicates statistical significance vs untreated, vehicle and histone culture cells ($P < 0.05$, $n = 3$). (**C**) Phase contrast pictures of HT1080 cells at 48 h. 1: untreated cells; 2: histone (2 μg); 3: rTIMP-3 (2 μg). The scale bar represents 100 μm and is applicable to all panels.

$n = 3$). Similar levels of TUNEL positivity were observed in HT1080 and MCF-7 cells (data not shown).

Induction of DNA synthesis by TIMP-3 was assessed in HeLa and HT1080 cells by BrdU analysis. TIMP-3 overexpression significantly elevated the number of cells in S phase compared to uninfected, RAd66-infected cultures and BB-94-treated cells (Figure 6, BB-94 data not shown).

To confirm deregulation of the cell cycle by TIMP-3, flow cytometric cell cycle analysis on propidium iodide-stained cells, HeLa and HT1080 cells was performed 42 h (HeLa) or 66 h (HT1080) post infection (Table 1). TIMP-3 overexpression resulted in a significant elevation in the number of cells in cycle, both in S and the $G_2$/M phases of the cell cycle with a corresponding reduction in $G_0/G_1$ (Table 1). In addition, by flow cytometry, the percentage of cells in pre-$G_0$ (apoptotic) was significantly elevated in RAdTIMP-3-infected cells compared to RAd66-infected controls (Table 1).

To examine whether the induction of apoptosis was initiated extracellularly, exogenous recombinant human TIMP-3 (rTIMP-3) was added to uninfected cultures of HT1080 cells. Western blot analysis revealed that exogenous TIMP-3 was associated with the cell/matrix lysates (Figure 7A). In comparison to vehicle and control protein-treated cultures, rTIMP-3 induced cell death (Figure 7B, C).

### DISCUSSION

The present study has documented the effect of overexpression of TIMP-3 on the invasion, proliferation and death of cancer cells in vitro. Efficient and high level TIMP-3 overexpression was demonstrated in HeLa cells whereas control adenoviral infection had no effect. Overexpression of TIMP-3 inhibited HeLa and HT1080 cell invasion through reconstituted basement membrane, an effect mimicked by overexpression of TIMP-1 and -2 and by a synthetic

© Cancer Research Campaign 1999

MMP inhibitor. TIMP-3 also promoted cell cycle entry observed by BrdU immunolabelling and by cell cycle analysis by flow cytometry. Overexpression of TIMP-3 culminated in apoptotic cell death of HeLa, HT1080 and MCF-7 cells assessed morphologically by flow cytometry and by TUNEL analysis. The effects on DNA synthesis and death mediated by TIMP-3 were not reproduced by a synthetic MMP inhibitor.

The inhibition of cancer cell invasion by TIMP-3 overexpression was not a surprising finding. TIMP-1 and -2 gene overexpression and a synthetic MMP inhibitor mimicked this effect consistent with previous data for TIMPs in vascular and cancer cells (DeClerck et al, 1992; Forough et al, 1996; Ahonen et al, 1998; Baker et al, 1998; George et al, 1998). TIMP-3 overexpression efficiently inhibited invasion of both the moderately invasive HeLa cell line and the highly invasive HT1080 cells. In other systems, mouse TIMP-3 overexpression in JB6 murine epithelial cells failed to inhibit invasion through reconstituted basement membrane or to affect in vivo tumorigenicity (Sun et al, 1996). These apparent discrepancies may be due to the differences in expression levels observed using plasmid- and adenoviral-based systems or it may represent different sensitivities to phenotypic alterations induced by TIMP-3 overexpression in tumour cells of different origins.

TIMP-3 overexpression was associated with induction of apoptotic cell death of HeLa, HT1080 and MCF-7 cells. This is consistent with our data in vascular smooth muscle cells and melanoma cells (Ahonen et al, 1998; Baker et al, 1998). Induction of cell death was associated with an elevation in the number of cells in S and $G_2$/M phases of the cell cycle determined by BrdU immunostaining and flow cytometry. In other systems, the chicken homologue of TIMP-3, ChIMP-3, induced DNA synthesis of transforming fibroblast cells resulting in detachment of the cells from the extracellular matrix (Yang and Hawkes, 1992). The ability of exogenously expressed TIMP-3 to induce DNA synthesis and apoptosis demonstrates a close link between initiation of cell proliferation and the induction of apoptosis in certain cell types. The endogenous TIMP-3 promoter has been shown to be tightly regulated during the $G_1$ phase of the cell cycle with promoter activity declining prior to S phase entry (Wick et al, 1994). It is therefore feasible that CMV IEP-driven TIMP-3 expression forces cells into the cell cycle. It has also been demonstrated that TIMP-3 overexpression through generation of stable colon carcinoma cell lines reduced tumour growth in nude mice and was associated with cell death by stabilization of tumour necrosis factor alpha (TNF-$\alpha$) receptors (Sun et al, 1996; Smith et al, 1997). Our data here and elsewhere (Baker et al, 1998) indicate that the TIMP-3-mediated induction of cell death is independent of MMP inhibitory activity as the effect is not mimicked by BB-94, a synthetic MMP inhibitor. The physiological involvement of TIMP-3 in the induction of cell death in the context of in vivo tumour development remains to be fully determined. High levels of TIMP-3 have been detected in breast carcinoma (Uría et al, 1994; Byrne et al, 1995) but the role of TIMP-3 in tumour invasion/growth remains to be determined. As high rates of apoptosis occur within tumour masses, an involvement for TIMP-3 could be postulated.

Our results have important implications for the role of TIMP-3 in physiological and pathological conditions and also for potential use of TIMP-3 gene overexpression on cancer gene therapy. TIMP-3 may have particular advantages over current cell death strategies as the protein is secreted and remains tightly bound to the extracellular matrix. This secreted TIMP-3 induces a bystander effect such that death of uninfected cells can occur. Additionally, TIMP-3-induced cell death does not require systemic delivery of a pro-drug, necessary for the thymidine kinase/gancyclovir cell death strategy. Further development of TIMPs in this context will clarify the role of MMPs in cancer progression. TIMP-3 appears to be an attractive candidate for future cancer gene therapy.

### ACKNOWLEDGEMENTS

The authors wish to thank Jason Johnson and Catherine Keen for technical assistance, and Clive Long, Pfizer (Sandwich, Kent, UK) for BB-94. This work was funded by the Medical Research Council for Great Britain.

### REFERENCES

Ahonen M, Baker AH and Kahari V-M (1998) Adenovirus-mediated delivery of tissue inhibitor of metalloproteinase-3 inhibits invasion and induces apoptosis in melanoma cells. *Cancer Res* **58**: 2310–2315

Apte SS, Hayashi K, Seldin MF, Mattei MG, Hayashi M and Olsen BR (1994) Gene encoding a novel murine tissue inhibitor of metalloproteinases (TIMP), TIMP-3, is expressed in developing mouse epithelia, cartilage and muscle, and is located on mouse chromosome-10. *Dev Dyn* **200**: 177–197

Baker AH, Wilkinson GWG, Hembry RM, Murphy G and Newby AC (1996) Development of recombinant adenoviruses that drive high level expression of the human metalloproteinase-9 and tissue inhibitor of metalloproteinase-1 and -2 genes: characterization of their infection into rabbit smooth muscle cells and human MCF-7 adenocarcinoma cells. *Matrix Biol* **15**: 383–395

Baker AH, Zaltsman AB, George SJ and Newby AC (1998) Divergent effects of tissue inhibitor of metalloproteinase-1, -2 or -3 overexpression on rat vascular smooth muscle cell invasion, proliferation and death in vitro: TIMP-3 promotes apoptosis. *J Clin Invest* **101**: 1478–1487

Bass KE, Li HX, Hawkes SP, Howard E, Bullen E, Vu TKH, McMaster M, Janatpour M and Fisher SJ (1997) Tissue inhibitor of metalloproteinase-3 expression is upregulated during human cytotrophoblast invasion in vitro. *Dev Genet* **21**: 61–67

Birkedal-Hansen H, Moore WGI, Bodden MK, Windsor LJ, Birkedal-Hansen B, DeCarlo A and Engler JA (1993) Matrix metalloproteinases: a review. *Crit Rev Oral Biol Med* **4**: 197–250

Bode W, Reinemer P, Huber R, Kleine T, Schnierer S and Tschesche H (1994) The X-ray crystal structure of the catalytic domain of human neutrophil collagenase inhibited by a substrate analogue reveals the essentials for catalysis and specificity. *EMBO J* **13**: 1263–1269

Byrne JA, Tomasetto C, Rouyer N, Bellocq JP, Rio MC and Basset P (1995) The tissue inhibitor of metalloproteinases-3 gene in breast carcinoma: identification of multiple polyadenylation sites and a stromal pattern of expression. *Mol Med* **1**: 418–427

DeClerck YA, Perez N, Shimada H, Boone TC, Langley KE and Taylor SM (1992) Inhibition of invasion and metastasis in cells transfected with an inhibitor of metalloproteinases. *Cancer Res* **52**: 701–708

Docherty AJP, Lyons A, Smith BJ, Wright EM, Stephens PE, Harris TJR, Murphy G and Reynolds JJ (1985) Sequence of human tissue inhibitor of metalloproteinases and its identity to erythroid-potentiating activity. *Nature* **318**: 66–69

Forough R, Koyama N, Hasenstab D, Lea H, Clowes M, Nikkari ST and Clowes AW (1996) Overexpression of tissue inhibitor of matrix metalloproteinase-1 inhibits vascular smooth muscle cell functions in vitro and in vivo. *Circ Res* **79**: 812–820

George SJ, Johnson JL, Angelini GD, Newby AC and Baker AH (1998) Adenovirus-mediated gene transfer of the human TIMP-1 gene inhibits SMC migration and neointima formation in human saphenous vein. *Hum Gene Ther* **9**: 867–877

Imren S, Kohn DB, Shimada H, Blavier L and DeClerck YA (1992) Overexpression of tissue inhibitor of metalloproteinases-2 by retroviral-mediated gene transfer in vivo inhibits tumor growth and invasion. *Cancer Res* **56**: 2891–2895

Koop S, Khokha R, Schmidt EE, MacDonald IC, Morris VL, Chambers AF and Groom AC (1994) Overexpression of metalloproteinase inhibitor in B16F10 cells does not affect extravasation but reduces tumor growth. *Cancer Res* **54**: 4791–4797

 © Cancer Research Campaign 1999

Leco KJ, Khokha R, Pavloff N, Hawkes SP and Edwards DR (1994) Tissue inhibitor of metalloproteinases-3 (TIMP-3) is an extracellular matrix-associated protein with a distinctive pattern of expression in mouse cells and tissues. *J Biol Chem* **269**: 9352–9360

Leco KJ, Apte SS, Taniguchi GT, Hawkes SP, Khokha R, Schultz GA and Edwards DR (1997) Murine tissue inhibitor of metalloproteinases-4 (TIMP-4): CDNA isolation and expression in adult mouse tissues. *FEBS Lett* **401**: 213–217

Low JA, Johnson MD, Bone EA and Dickson RB (1996) The matrix metalloproteinase inhibitor Batimastat (BB-94) retards human breast cancer solid tumor-growth but not ascites formation in nude mice. *Clin Cancer Res* **2**: 1207–1214

Montgomery AMP, Mueller BM, Reisfeld RA, Taylor SM and Declerck YA (1994) Effect of tissue inhibitor of the matrix metalloproteinases-2 expression on the growth and spontaneous metastasis of a human melanoma cell line. *Cancer Res* **54**: 5467–5473

Murphy G, Willenbrock F, Ward RV, Cockett MI, Eaton D and Docherty AJP (1992) The C-terminal domain of 72 kDa gelatinase A is not required for catalysis, but is essential for membrane activation and modulates interactions with tissue inhibitors of metalloproteinases. *Biochem J* **283**: 637–641

Noel AC, Calle A, Emonard HP, Nusgens BV, Simar L, Foidart J, Lapiere CM and Foidart JM (1991) Invasion of reconstituted basement-membrane matrix is not correlated to the malignant metastatic cell phenotype. *Cancer Res* **51**: 405–414

Nuovo GJ (1997) In situ detection of PCR-amplified metalloproteinase cDNAs, their inhibitors and human papillomavirus transcripts in cervical carcinoma cell lines. *Int J Cancer* **71**: 1056–1060

Sledge GW, Qulali M, Goulet R, Bone EA and Fife R (1995) Effect of matrix metalloproteinase inhibitor Batimastat on breast cancer regrowth and metastasis in athymic mice. *J Natl Cancer Inst* **87**: 1546–1550

Smith MR, Kung HF, Durum SK, Colburn NH and Sun Y (1997) TIMP-3 induces cell death by stabilizing TNF-α receptors on the surface of human colon carcinoma cells. *Cytokine* **9**: 770–780

Stetler-Stevenson WG, Brown PD, Onisto M, Levy AT and Liotta LA (1990) Tissue inhibitor of metalloproteinases-2 (TIMP-2) mRNA expression in tumor cell lines and human tumor tissues. *J Biol Chem* **265**: 13933–13938

Strongin AY, Collier I, Bannikov BL, Grant GA and Goldberg GI (1995) Mechanism of cell surface activation of 72 kDa type IV collagenase. *J Biol Chem* **270**: 5331–5338

Sun Y, Kim H, Parker M, Stelerstevenson WG and Colburn NH (1996) Lack of suppression of tumor-cell phenotype by overexpression of TIMP-3 in mouse Jb6 tumor-cells: identification of a transfectant with increased tumorigenicity and invasiveness. *Anticancer Res* **16**: 1–7

Uría JA, Ferrando AA, Velasco G, Freije JMP and López-Otín C (1994) Structure and expression in breast tumors of human TIMP-3, a new member of the metalloproteinase inhibitor family. *Cancer Res* **54**: 2091–2094

Wang X, Fu X, Brown PD, Crimmin MJ and Hoffman RM (1994) Matrix metalloproteinase inhibitor BB-94 (Batimastat) inhibits human colon tumor growth and spread in a patient-liek orthotopic model in nude mice. *Cancer Res* **54**: 4726–2728

Wang MS, Liu YLE, Greene J, Sheng SJ, Fuchs A, Rosen EM and Shi YE (1997) Inhibition of tumor growth and metastasis of human breast cancer cells transfected with tissue inhibitor of metalloproteinase 4. *Oncogene* **14**: 2767–2774

Watanabe M, Takahashi Y, Ohta T, Mai M, Sasaki T and Seiki M (1996) Inhibition of metastasis in human gastric cancer cells transfected with tissue inhibitor of metalloproteinase-1 gene in nude mice. *Cancer* **77**: 1676–1680

Watson SA, Morris TM, Parsons SL, Steele RJC and Brown PD (1996) Therapeutic effect of the matrix metalloproteinase inhibitor, Batimastat, in a human colorectal cancer ascites model. *Br J Cancer* **74**: 1354–1358

Wick M, Burger C, Brusselbach S, Lucibello FC and Muller R (1994) A novel member of human tissue inhibitor of metalloproteinases (TIMP) gene family is regulated during G1 progression, mitogenic stimulation, differentiation and senescence. *J Biol Chem* **269**: 18953–18960

Wilkinson GWG and Akrigg A (1992) Constitutive and enhanced expression from the CMV major IE promoter in a defective adenovirus vector. *Nucleic Acids Res* **20**: 2233–2239

Yang T and Hawkes SP (1992) Role of the 21-kDa protein TIMP-3 in oncogenic transformation of cultured embryo fibroblasts. *Proc Natl Acad Sci USA* **89**: 10676–10680

© Cancer Research Campaign 1999
